# Endovascular recanalization of symptomatic non-acute occlusion of the vertebrobasilar artery

**DOI:** 10.3389/fneur.2023.1125244

**Published:** 2023-04-14

**Authors:** ZhiLong Zhou, TianXiao Li, LiangFu Zhu, LiHeng Wu, Min Guan, ZhenKai Ma, YangHui Liu, Jin Qin, BuLang Gao

**Affiliations:** Stroke Center, People's Hospital of Zhengzhou University, Henan Provincial People’s Hospital, School of Clinical Medicine, Henan University, Zhengzhou, Henan, China

**Keywords:** intracranial vertebral artery, basilar artery, symptomatic occlusion, endovascular recanalization, non-acute

## Abstract

**Purpose:**

The study aimed to investigate the safety, effect, and risk factors of endovascular recanalization of symptomatic non-acute occlusion of the vertebrobasilar artery (SNOVA).

**Materials and methods:**

Patients with SNOVA were retrospectively enrolled and treated with endovascular recanalization. The clinical data, endovascular treatment, peri-procedural complications, and follow-up outcomes were analyzed.

**Results:**

A total of 88 patients were enrolled, with an interval to recanalization of 2–89 days (median 23) and an mRS of 2–5 (median 3 and IQR 1). Occlusion was in the intracranial vertebral artery in 68 (77.27%) patients and basilar artery in 20 (22.73%), with an occlusion length of 4.5–43.7 mm (mean 18.3 ± 8.8). Endovascular recanalization was successful in 81 (92.0%) patients. Post-dilatation was performed in 23 (28.4%) patients. After stenting, the residual stenosis was 10%–40% (mean 20.2% ± 7.6%). Peri-procedural complications occurred in 17 (19.3%) patients, with a mortality rate of 5.7%. In total, 79 (95.18%) patients underwent follow-up 5–29 (mean 16.9 ± 5.5) months later, with an mRS score of 0–6 (median 1 and IQR 1) at follow-up, being significantly (*p* < 0.0001) better than that at discharge. Stroke occurred in 9 patients (11.4%) in 1 year. In-stent restenosis occurred in 19 (25.33%) patients. Significant (*p* < 0.05) independent risk factors were blunt occlusion for successful recanalization, duration to recanalization and blunt occlusion for peri-procedural complications, and post-dilatation for both in-stent restenosis and 1-year stroke or death events.

**Conclusion:**

Endovascular recanalization of symptomatic non-acute occlusion of the vertebrobasilar artery is feasible even for a long occlusion segment, with a high recanalization rate, a low complication rate, and a good prognosis. Blunt occlusion and duration from the onset to recanalization may affect successful recanalization and peri-procedural complications while post-dilatation may affect in-stent restenosis and prognosis.

## Introduction

The intracranial vertebral artery and the basilar artery are the common locations of atherosclerotic occlusion (1–7), and after acute occlusion, some patients may survive and continue to suffer from repeated transient ischemic attacks and strokes in the non-acute or chronic stage even under aggressive medication (8–10). Symptomatic non-acute intracranial vertebrobasilar artery occlusion (VBAO) is often accompanied by insufficient collateral circulation to compensate for blood supply, and there will still be a high incidence of ischemic events and subsequent disastrous outcomes even with the best medical treatment (11, 12). In recent years, with the improvement of the concept of ischemic prevention and treatment and the progress of endovascular instruments, endovascular treatment of non-acute occlusion of intracranial arteries has been increasingly performed, but its perioperative complications and mortality remain high (8.3%–44.4%) (11–15). With the accumulation of experience in endovascular recanalization of VBAO, better outcomes may be achieved. This study aimed to investigate the safety and complications of endovascular recanalization of symptomatic non-acute VBAO and risk factors for the prognosis in a cohort of patients with symptomatic non-acute VBAO at a high-volume medical center.

## Materials and methods

### Subjects

**Figure 1:**
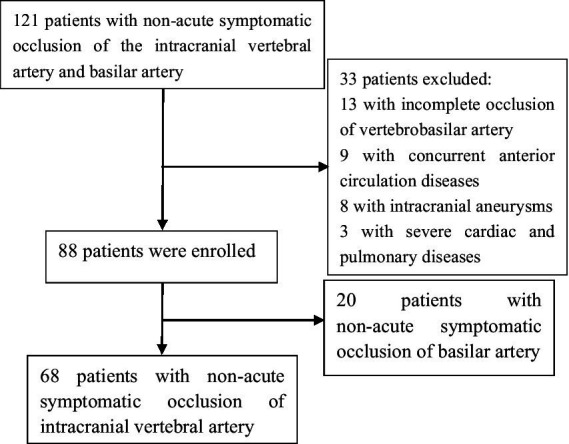
Flowchart of patient enrollment.

This retrospective one-center study was performed after being approved by the ethics committee of our hospital, and all patients had given signed informed consent to participate. Between July 2017 and June 2022, patients with symptomatic non-acute occlusion of the intracranial vertebral artery and basilar artery treated with endovascular recanalization were enrolled. The inclusion criteria were consecutive patients with symptomatic non-acute occlusion of the intracranial vertebral and basilar artery; confirmation of intracranial arterial occlusion by digital subtraction cerebral angiography; 0 in the thrombolysis of the cerebral infarction (TICI) grade; occlusion duration ranging from 24 h to 3 months after symptom onset; MRI-confirmed cerebral infarction in the vertebrobasilar artery distribution area involving the pons, cerebellum, cerebral peduncle, occipital lobe, and thalamus; and repeated transient ischemic attacks or strokes in the posterior circulation even after the best medical treatment. The exclusion criteria were patients with non-atherosclerotic occlusion of the intracranial vertebral and basilar artery; severe calcification in the atherosclerotic lesions confirmed on medical imaging; allergies to heparin, aspirin, clopidogrel, metal implants, or narcotic drugs; intolerance to general anesthesia, comorbidities of malignant tumor or severe liver and kidney dysfunction; combined intracranial aneurysms or anterior circulation diseases; and an expected life span of less than 1 year (Figure 1).

Hypertension, diabetes mellitus, and hyperlipidemia were diagnosed according to the recommended criteria in the guidelines (16, 17). Alcohol abuse and smoking were assessed using the following criteria (18): more than 182 and 117 g of pure alcohol per week on average for male patients and female patients, respectively, and more than one cigarette a day on average for both sexes. The independent living ability of patients was evaluated with the modified Rankin Scale (mRS) score (19).

### Endovascular procedures

Before endovascular recanalization, clopidogrel (75 mg, once a day) combined with aspirin (100 mg, once a day) was administered for ≥3 days for all patients. All endovascular procedures were performed under general anesthesia. The Seldinger technique was used to puncture the right femoral artery before inserting a 6F sheath. Thereafter, unfractionated heparin at 50 IU/kg was injected intravenously for systemic heparinization. During the recanalization procedure, 1,000 IU of unfractionated heparin was added intravenously every hour. After a 6-F guiding catheter was placed at the vertebral artery proximal to the occluded location, an Excelsior SL-10 microcatheter (Stryker, Fremont, CA, USA) was carefully passed through the occluded segment under the guidance of a 0.014 inch (0.036 cm) micro-guide wire with a length of 200 cm (Asahi, Nagoya, Aichi, Japan). Thereafter, an ASAHI 300 cm micro-guide wire was exchanged and introduced into the relevant artery before the selection of a Gateway balloon with a diameter of 80% of the normal vessel diameter (2.0 mm × 15 mm or 2.5 mm × 15 mm, Stryker, Fremont, CA, USA) for pre-dilatation. An appropriate Enterprise stent (Codman & Shurtleff, Raynham, MA, USA) was chosen and placed at the occluded segment with the stent of size 3–5 mm longer than either the proximal or distal end of the occluded segment. If the available stent was not long enough to completely cover the occluded segment, a self-expandable or balloon-expandable stent was used to overlap the previously deployed stent and cover the occluded segment. If the residual stenosis was >50% after stenting, post-stenting dilatation was performed with a balloon.

After recanalization, the forward blood flow was evaluated according to the TICI grading system, and TICI ≥2b was defined as successful recanalization of blood vessels. The patient was instructed to stay in bed with monitoring of neurological function. Blood pressure was strictly controlled, with the systolic pressure being maintained at 110–130 mmHg within 24–48 h after recanalization. Head CT was rechecked within 24 h. Usually, the patient was discharged from the hospital 5 days after recanalization. Clopidogrel (75 mg, once a day) and aspirin (100 mg, once a day) were routinely administered for 6 months before when aspirin (100 mg, once a day) alone was taken orally for a long time.

### Follow-up

Follow-up was conducted by telephone and outpatient or inpatient reexamination. The clinical follow-up time was 1 week, 6 months, and 1 year after the procedure, and all neurological and non-neurological complications were assessed. The patients were scored with the mRS before and after recanalization and during follow-up. Digital subtraction angiography was performed 6 months after recanalization, and restenosis was defined as a stenosis of ≥50% in the stent (20).

### Parameters of evaluation

**Figure 2:**
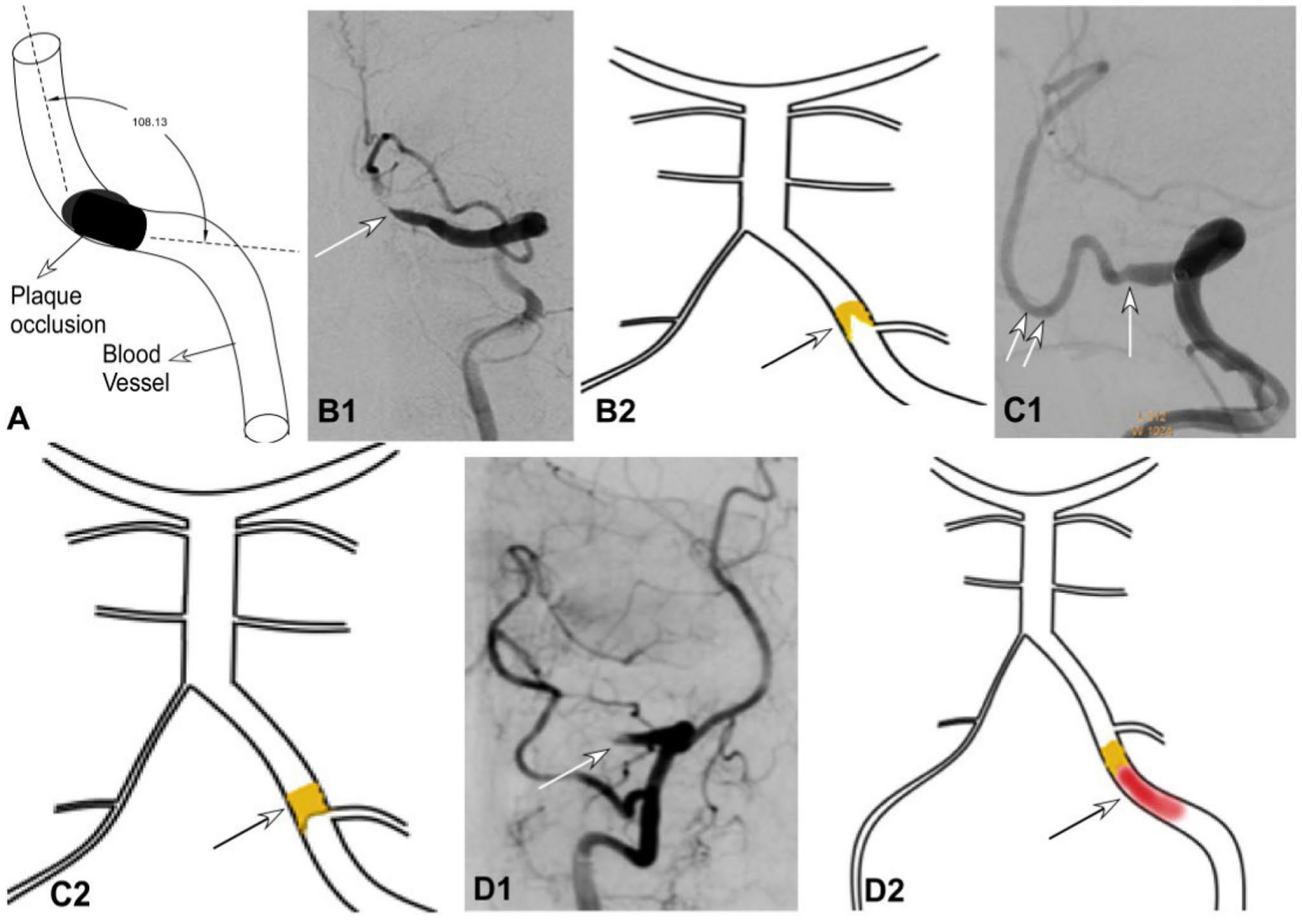
Occlusion angles and types of occlusion shape. **(A)** The arterial angle formed between the proximal and distal segments at the occlusion was measured. **(B–D)** Types of occlusion shape were defined, with the sharp occlusion in **B1**, **B2** (arrow), blunt occlusion in **C1**, **C2** (longer arrow), and flame-like occlusion in **D1, D2** (arrow). The double arrows in **C** indicate the posterior inferior cerebellar artery. (**B1**, **B2**) The sharp occlusion shape in the major blood-supplying vertebral artery was shown in cerebral angiography in **B1** and in the sketch in **B2**. (**C1**, **C2**) The blunt occlusion in the major blood-supplying vertebral artery was shown in cerebral angiography in **C1** and in the sketch in **C2**. (**D1**, **D2**) The flame-like occlusion in the major blood-supplying vertebral artery was shown in cerebral angiography in **D1** and in the sketch in **D2**.

The following parameters were evaluated, including the patient’s age, sex, past history, smoking, alcohol abuse, location of occlusion, length and angle of occlusion that was measured after stenting (Figure 2A), types of occlusion, time from symptom onset to recanalization, successful recanalization or not, time for the micro-guide wire to pass the occlusion, total surgical time, the number and types of balloons and stents used in the procedure, residual stenosis, peri-procedural complications, follow-up time and outcomes, in-stent restenosis, and 1-year stroke rate. A total of three types of occlusions were defined based on the morphology of the occlusion: type 1 sharp occlusion, type 2 blunt occlusion, and type 3 flame-like occlusion (Figures 2B1–D2).

In measuring the length of the occluded segment, a 5F 115 cm intermediate catheter was first navigated to the beginning of the intracranial segment of the vertebral artery before performing angiography (with the contrast agent injection pressure: 300 psi, 3 ml/s, and a total volume: 7 mL). At the end of angiography (20 s), the occlusion position displayed was the proximal end of the occlusion, and the compensatory arterial segment displayed at the distal end was the distal end of the occlusion. Postoperative 3-dimensional rotational angiography was performed to accurately measure the length of the proximal and distal end of the occlusion which was determined before the operation.

### Statistical analysis

Statistical analysis was conducted with the SPSS 17.0 software (IBM, Chicago, IL, USA). Continuous measurement data were presented as mean ± standard deviation if in the normal distribution and tested with the paired *t*-test as the median and interquartile range (IQR) if not in the normal distribution and also tested with the chi-square test. Classification data were expressed in frequency and percentage terms and Wilcoxon rank sum test was used. Univariate logistic regression analysis was used to analyze risk factors for successful recanalization, residual stenosis, peri-procedural complications, poor follow-up outcomes (mRS), in-stent restenosis, and 1-year stroke or death events, and parameters with a *p*-value of ≤0.1 were used in the multivariate logistic analysis for independent risk factors. The odds ratio (OR) and 95% confidence interval (CI) were calculated. Continuous measurement data in the logistic analysis were used in the receiver operating character (ROC) curve analysis. The statistically significant *p*-value was set at <0.05.

## Results

### Subjects

A total of 88 patients were enrolled, including 72 male and 16 female patients aged 37–82 years (mean 58.5 ± 9.0) (Figure 1; Table 1). The duration from symptom onset to recanalization was 2–89 days (median 23). Occlusion was in the intracranial vertebral artery in 68 (77.27%) patients and basilar artery in 20 patients (22.73%), with an occlusion length of 4.5–43.7 mm (mean 18.3 ± 8.8). The occlusion length was significantly longer in patients with the vertebral artery occlusion than those with the basilar artery occlusion (21.11 ± 7.9 mm vs. 8.88 ± 3.50 mm, *p* < 0.0001). The arterial angle formed between the segments proximal and distal to the occlusion was 56–176° (mean 148.8° ± 19.4°), and the mRS was 2–5 (mean 3.3 ± 1.1, median 3, and IQR 1) at admission.

**Table 1:** Clinical data of patients.

Variables	Total (*n* = 88)	Vertebral artery occlusion (*n* = 68)	Basilar artery occlusion (*n* = 20)	*p*
M/F	72/16	58/10	14/6	0.14
Age (y)	37–82 (58.5 ± 9.0)	37–82 (58.9 ± 9.1)	48–73 (57.4 ± 8.5)	0.53
Hypertension	70 (79.5%)	54 (79.4%)	16 (80.0%)	0.95
Diabetes mellitus	32 (36.4%)	24 (35.3%)	8 (40.0%)	0.70
Coronary heart disease	12 (13.6%)	8 (11.8%)	4 (20.0%)	0.36
Hyperlipidemia	26 (29.5%)	20 (29.4%)	6 (30.0%)	0.96
HCY	17 (19.3%)	13 (19.1%)	4 (20.0%)	0.93
Smoking	49 (55.7%)	39 (57.4%)	10 (50.0%)	0.56
Alcohol abuse	39 (44.3%)	30 (44.1%)	9 (45.0%)	0.94
Duration from onset to surgery (d)	2–89 (23, 19.5)	2–75 (23, 20.75)	3–89 (24, 19.5)	0.39
mRS before surgery	2–5 (3.30 ± 1.06)	2–5 (3.34 ± 1.13)	2–5 (3.15 ± 0.81)	0.49
mRS at discharge	1–6 (2.64 ± 1.27)	1–5 (2.60 ± 1.20)	1–6 (2.79 ± 1.51)	0.57
mRS at 6 months	0–6 (1, 1)	1 (1, 1)	1 (1, 2.75)	0.16
Sharp occlusion	42 (47.7%)	30 (44.1%)	12 (60.0%)	0.10
Blunt occlusion	22 (25.0%)	16 (23.5%)	6 (30.0%)	
flame-like occlusion	24 (27.3%)	22 (32.4%)	2 (10.0%)	
Occlusion length (mm)	4.5–43.7 (18.3 ± 8.8)	6.5–43.7 (21.11 ± 7.96)	4.5–15.2 (8.88 ± 3.50)	<0.0001
Occlusion angle(°)	56°–176° (148.8 ± 19.4°)	56–176 (147.47 ± 20.43)	127–172 (154 ± 14.46)	0.21

HCY, hyperhomocysteinemia; mRS, modified Rankin Scale score; P, comparison between patients with intracranial vertebral artery occlusion and those with basilar artery occlusion. Data presentation: continuous data were presented as mean ± standard deviation if in the normal distribution but as the median and interquartile range (IQR) if not in the normal distribution.

### Endovascular treatment

Endovascular recanalization was successful in 81 (92.0%) patients, resulting in a TICI grade 3 reperfusion in all patients (Table 2). The time for a micro-guide wire to pass through the occluded segment was 0.3–52 min (median 3 and IQR 9), and the total surgical time was 60–350 min (mean 149.7 ± 45.5). A total of 110 stents were deployed, including 83 (75.5%) Enterprise stents, 21 (19.1%) Apollo (MicroPort, Shanghai, China), three (2.7%) Lvis (MicroVention, Tustin, California, USA), two (1.8%) Resolute (Medtronic, Irvine, CA, USA), and one (0.9%) Neuroform (Stryker, Kalamazoo, MI, USA). One stent was used in 54 patients each, two stents in 27, and three stents in two. The Enterprise stent was used in 80 patients while in one patient, a Lvis, an Apollo, and a Resolute stent were used.

**Table 2:** Data of endovascular recanalization.

Variables	Total (*n* = 88)	Vertebral artery occlusion (68)	Basilar artery occlusion (*n* = 20)	*p*
Success rate	81 (92.0%)	63 (92.6%)	18 (90%)	0.57
Time for the guide wire to pass occlusion (min)	0.3–52 (3, 9)	0.5–52 (3.5, 11)	0.3–47 (2, 3.48)	0.87
Total surgical time (min)	60–350 (149.7 ± 45.5)	60–350 (149.12 ± 46.60)	85–240 (151.5 ± 42.86)	0.84
Balloon diameters	1.5–3 (2.18 ± 0.27)	2–3 (2.23 ± 0.27)	1.5–2.5 (2 ± 0.16)	0.0005
Post-dilatation	23 (26.1%)	20 (29.4%)	3 (15%)	0.18
Residual stenosis	10%–40% (20.2% ± 7.6%)	10%–40% (20.5% ± 7.7%)	10%–30% (19.3% ± 7.5%)	0.51
Complications	17 (19.3)	11 (16.2%)	6 (30%)	0.33
Dissection	7 (7.95%)	5 (7.35%)	2 (10%)	
Bleeding	2 (2.27%)	0	2 (10%)	
Acute thrombosis	6 (6.82%)	4 (5.88%)	2 (10%)	
Failed recanalization	7 (7.95%)&	5 (7.35%)&	2 (10%)#	
Mortality	7 (8.0%)	1 (1.5%)	4 (20%)	0.06
Follow-up time (m)	5–29 (16.9 ± 5.5)	5–27 (16.56 ± 5.17)	6–29 (18.2 ± 6.96)	0.31
Patients with clinical follow-up (n)	79 (95.18%)	64 (94.12%)	15 (75%)	0.57
Patients with angiographic follow-up	75 (90.36% or 75/83)	61 (95.31% or 61/64)	14 (93.33% or 14/15)	0.27
mRS at discharge	1–6 (2.64 ± 1.27)	1–5 (2.60 ± 1.20)	1–6 (2.79 ± 1.51)	0.57
6-month mRS	0–6 (1, 1)**	0–6 (1.60 ± 0.19)**	0–6 (2.47 ± 0.35)	0.033
In-stent restenosis	19 (25.3% or 19/75)	17 (27.9% or /61)	2 (14.3% or 2/14)	0.27
1-year stroke	9 (13.0% or 9/69)	8 (14.0% or 8/57)	1 (8.3% or 1/12)	0.58

*p* indicates comparison between patients with vertebral and basilar artery occlusion; mRS, modified Rankin Scale score; Data presentation: continuous data were presented as range and mean ± standard deviation if in the normal distribution but as the median and interquartile range (IQR) if not in the normal distribution. ** < 0.0001 compared with mRS at discharge; three of the failed patients in the vertebral artery had dissection, and # two failed patients in the basilar artery had dissection.

Post-dilatation after stenting was performed in 23 (28.4% or 23/81) patients, and the residual stenosis was 10–40% (mean 20.2% ± 7.6%). At discharge, the mRS was 1–6 (mean 2.6 ± 1.2, median 2 and IQR 1), which is significantly (*p* < 0.0001) better than that at admission.

In 68 patients with vertebral artery occlusion, recanalization was successful in 63 (92.6%) patients but failed in five (7.4%). Among the failed patients, two patients successfully underwent Enterprise stent angioplasty of the contralateral inferior vertebral artery for remedy while the other three patients were treated with medications conservatively. Among 63 patients with successful recanalization, the arteries for major blood supply in 62 (98.4%) patients were recanalized, with 33 (52.4%) patients being treated with the Enterprise stent angioplasty, 29 (46.0%) treated with the Enterprise stent combined with a balloon-expandable stent, and one (1.6%) with balloon-expandable stents. Overlapped stents were used in 29 (46.0%) patients while a long Enterprise stent (28 mm or 37 mm) was used in 44 (69.8%) patients. Peri-procedural complications took place in 11 (16.2%) patients including five (7.4%) cases with unsuccessful recanalization. Overall, five (7.4%) patients experienced arterial dissection, and the dissection in three patients resulted in failed recanalization, with the dissection in the other two patients being treated with stenting. Among four (5.88%) patients with intraprocedural thrombosis, one (1.5%) patient with distal thrombosis in the basilar artery died, and the other three (4.41%) patients improved after thrombolysis.

In 20 patients with basilar artery occlusion, recanalization was successful in 18 (90%) patients. In total, two (10%) patients failed, including one with dissection and one with bleeding who both died during the peri-procedural period. All 18 patients were recanalized using the Enterprise stent angioplasty. Complications occurred in six (30%) patients including two patients with unsuccessful recanalization. One patient experienced acute thrombosis which was successfully treated with intra-arterial thrombolysis and thrombectomy, one patient had dissection at the procedure and died 15 days later, and one patient experienced pontine hemorrhage and died 1 week later. The death rate was 20% (4/20).

### Complications

Peri-procedural complications occurred in 17 (19.3%) patients, including 11 (16.2%) patients in recanalizing the vertebral artery and six (30%) in recanalizing the basilar artery (Table 2). A total of five patients died during the peri-procedural period, resulting in a mortality of 5.7%, with one death (1.1%) in recanalizing the vertebral artery and four (4.5%) in recanalizing the basilar artery.

### Occlusion length and shape

The occlusion length was not significantly different between patients with and without complications (19.15 ± 9.11 mm vs. 18.13 ± 8.57 mm, *p* = 0.67) or successful recanalization (18.15 ± 8.76 mm vs. 20.39 ± 10.13 mm, *p* = 0.52). Patients with blunt occlusion experienced a significantly (*p* < 0.05) longer duration for the micro-guide wire to pass through the occlusion than those with sharp (median 25.5 vs. 2 min, *p* < 0.0001) or flame-like occlusion (median 25.5 vs. 3.5 min, p < 0.0001) (Table 3). The total surgical time was also significantly (p < 0.05) greater in patients with blunt occlusion than those with sharp occlusion (166.36 ± 45.49 vs. 139.64 ± 49.29 min, *p* = 0.039). In seven patients without successful recanalization, the failure occurred in blunt occlusion in six (85.71%) patients, but in sharp occlusion, failure occurred only in only one (14.29%) patient. Patients with flame-like occlusion were all successfully recanalized.

**Table 3:** Comparison of data in different shapes of occlusion.

Occlusion shape	No.	Occlusion length (mm)	Occlusion angle (°)	Duration to pass occlusion (min)	Total surgical time (min)	Successful recanalization rate
Sharp	42	4.5–43.7 (15.76 ± 8.31)	56–176 (149.74 ± 23.08)	0.3–28 (2, 1.775) **	75–350 (139.64 ± 49.29)*	41 (97.62%)
Blunt	22	5.5–35 (20.21 ± 9.81)	102–172 (148 ± 16.18)	1–52 (25.5, 27)	60–240 (166.36 ± 45.49)	16 (72.73%)
Flame-like	24	9–42 (21.09 ± 7.80)	110–165 (147.96 ± 15.13)	0.5–23 (3.5, 4.5)**	60–210 (151.88 ± 34.19)	24 (100%)

*p-value < 0.05 compared with blunt occlusion; **p < 0.001 compared with blunt occlusion; Data presentation: continuous data were presented as mean ± standard deviation if in the normal distribution but as median and interquartile range (IQR) if not in the normal distribution.

### Follow-Up

Among 83 (94.32%) patients who were alive after recanalization, 79 (95.18% or 79/83) with successful recanalization underwent follow-up 5–29 (mean 16.9 ± 5.5) months later (Table 2), with an mRS score of 0–6 (median 1 and IQR 1) at follow-up, which was significantly (*p* < 0.0001) better than that at discharge. Patients with the vertebral artery occlusion had a significantly (*p* = 0.033) better mRS than patients with the basilar artery occlusion (1.60 ± 0.19 vs. 2.47 ± 0.35). Stroke occurred in nine patients at 1 year resulting in a 1-year stroke rate of 11.4% (9/79). Among 75 (90.36%, 75/83) patients with angiographic follow-up, in-stent restenosis occurred in 19 (25.33% or 19/75) patients.

### Logistic regression analysis and ROC curve analysis

In univariate logistic regression analysis, duration from disease onset to recanalization (*p* = 0.015, OR 7.67, 95% CI 1.52–38.65) and blunt occlusion (*p* = 0.0004, OR 24.38, 95% CI 2.74–217.04) were risk factors for failing recanalization, whereas diabetes mellitus (*p* = 0.004, OR 5.24, and 95% CI 1.62–16.94), duration to recanalization (*p* = 0.001, OR 7.88, 95% CI 2.31–26.82), and blunt occlusion (p = 0.0004, OR 8.19, 95% CI 2.50–26.86) were risk factors for peri-procedural complications (Table 4). Blunt occlusion was a significant (*p* = 0.003, OR 5.18, 95% CI 1.73–15.50) risk factor for poor follow-up mRS outcomes (mRS > 2), whereas post-dilatation was a risk factor for in-stent restenosis (*p* = 0.007, OR 4.55, 95% CI 1.49–13.88) and 1-year stroke or death events (*p* = 0.016, OR 6, 95% CI 1.33–27.00). In multivariate logistic regression analysis, significant (*p* < 0.05) independent risk factors were blunt occlusion for failing recanalization, duration to recanalization, and blunt occlusion for peri-procedural complications, and blunt occlusion was the only independent risk factor for both the in-stent restenosis and 1-year stroke or death events (Table 4).

**Table 4:** Univariate and multivariate logistic analyses for risk factors.

Dependent variables	Independent variables	Univariate analysis				Multivariate analysis	
		Statistical value	*p*	OR	95%CI	Statistical value	*p*
Successful recanalization	Duration to recanalization	5.93	0.015	7.67	1.52–38.65		
	Blunt occlusion	12.72	0.0004	24.38	2.74–217.04	7.57	0.006
Complications	Diabetes	8.35	0.004	5.24	1.62–16.94		
	Duration to recanalization>43d	10.87	0.001	7.88	2.31–26.82	4.96	0.026
	Blunt occlusion	12.71	0.0004	8.19	2.50–26.86	5.94	0.015
mRS outcome	Blunt occlusion	8.73	0.003	5.18	1.73–15.50		
In-stent restenosis	Post-dilatation	7.17	0.007	4.55	1.49–13.88	5.00	0.025
1-year stroke or death	Post-dilatation	5.86	0.016	6.00	1.33–27.00	5.01	0.025

OR, odds ratio; CI, confidence interval; mRS, modified Rankin Scale.

**Figure 3:**
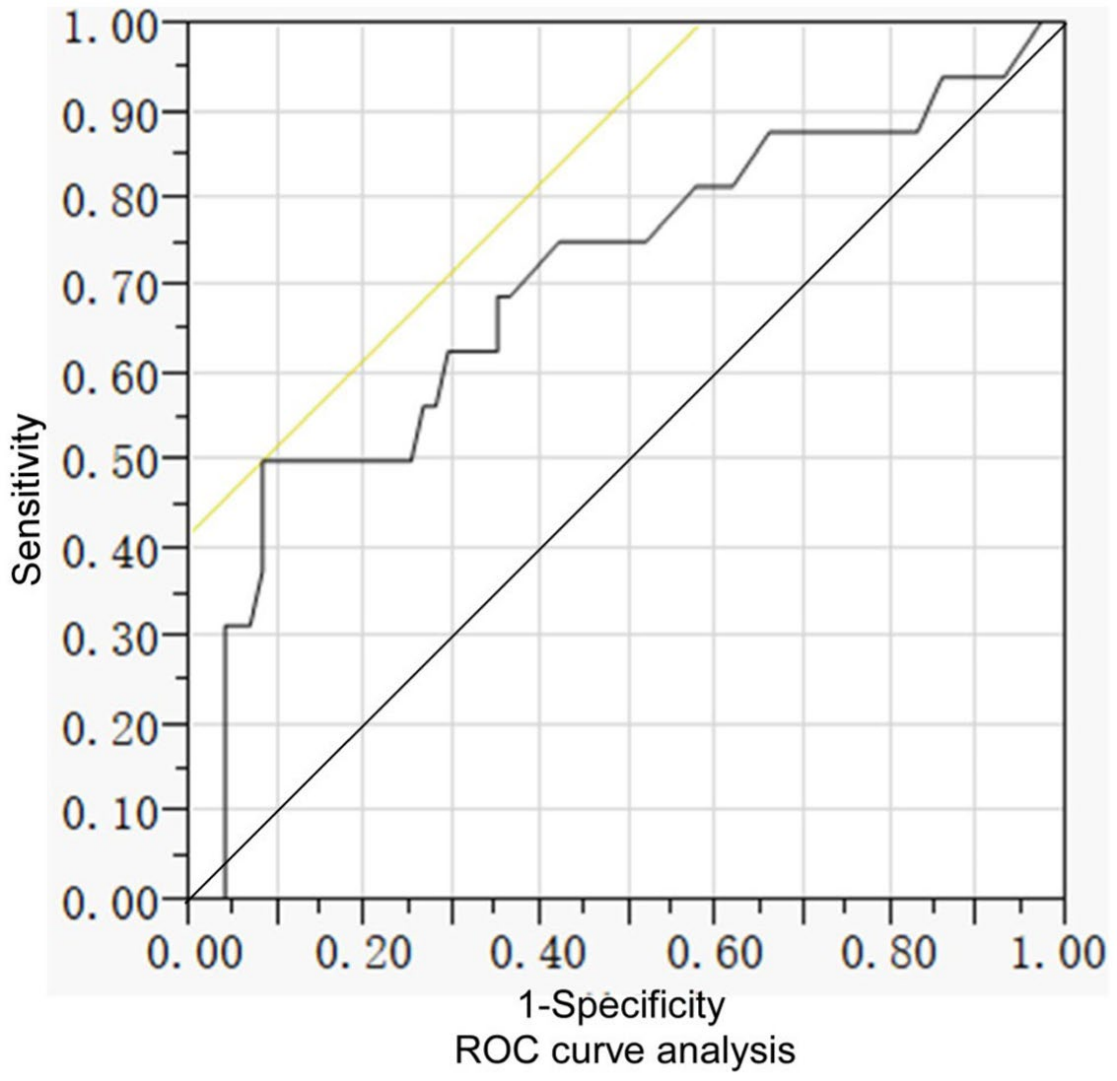
Receiver operating characteristic (ROC) curve analysis of the duration from symptom onset to recanalization as a risk factor for peri-procedural complications.

ROC curve analysis revealed that the duration from symptom onset to recanalization was a risk factor for peri-procedural complications with an area under the curve of 0.70, a cutoff value of 45 days, a sensitivity of 0.50, a specificity of 0.92, a positive predictive value of 0.57, and a negative predictive value of 0.89 (Figure 3).

## Discussion

In this study, it was found that endovascular recanalization for symptomatic non-acute occlusion of the vertebrobasilar artery was feasible even for a long occlusion, with a high recanalization rate, high safety, and good prognosis. Blunt occlusion was an independent risk factor for successful recanalization; blunt occlusion and duration to recanalization were independent risk factors for peri-procedural complications; and post-dilatation was an independent risk factor for in-stent restenosis and 1-year stroke or death events.

In order to improve the opening rate of VBAO, we focused on the evaluation of the occlusion by preoperative CT angiography or digital subtraction angiography, including the occlusion time, occlusion proximal morphology, and length. Currently, symptomatic occlusion within 24 h is considered acute occlusion. In this study, non-acute occlusion is defined as symptomatic occlusion occurring >24 h. In the study of Mori et al. (21), four occlusive patients received reperfusion within less than 3 months. It was thus believed that thrombus calcification and organization were relatively serious at the occlusion location when occlusion time was over 3 months, leading to a difficult opening. Patients in our study were selected with an occlusion time of less than 3 months. Further analysis of our data revealed that duration from symptom onset to recanalization significantly affects successful recanalization and peri-procedural complications, with the cutoff value of duration to recanalization as 45 days with an area under the curve of 0.70.

In endovascular recanalization of the VBAO, the primary technical challenge is to traverse the occlusion location with a micro-guide wire. Some authors thought that pre-surgical high-resolution MRI and simultaneous two-artery angiography during the endovascular recanalization procedure might be useful in guiding the guide wire through the occlusion to the distal arterial lumen (22). Others believed that the patient’s complaint of pain, sweating, and vomiting during the procedure may indicate arterial perforation by the micro-guide wire, so timely communication with the patient during recanalization may be helpful to decide whether the guide wire is located in the arterial true lumen.

In our study, the occlusion shape was classified into three types. The sharp occlusion was the beginning of an atherosclerotic occlusive segment, with a relatively short occlusive time. This type of occlusion had a tip to stabilize the micro-guide wire and increase its ability to “drill” into the occlusion location and the subsequent probability of recanalization. In our study of 42 patients with sharp occlusion, only one (2.38%) failed. The blunt occlusion had a long occlusive time with the formation of a thick strong fibrous cap which may indicate the beginning of atherosclerosis. Among 22 patients with blunt occlusion, six (27.27%) failed to be opened, accounting for 85.71% of all seven patients without successful recanalization. This may indicate that patients with blunt occlusion may not be easily recanalized because the recanalized patients with this type of occlusion also experienced significantly longer surgical time and longer duration for the guide wire to pass through the occlusion than those with sharp occlusion. Moreover, in one patient with blunt occlusion of the intracranial vertebral artery, recanalization initially failed but was successful by reverse opening *via* the contralateral vertebral artery. In the flame-like occlusion, the true atherosclerotic-occluded location was at the distal end. Because of no outflow tract of side branches, the blood flow proximal to the real occlusion was stagnant with rich thrombi formed. This type of occlusion was also easily recanalized, and all 24 patients with this type of occlusion had got complete recanalization.

In recanalizing VBAO, the occlusion length was not particularly important such as the occlusion shape. Although the occlusion was significantly longer in the intracranial vertebral artery than in the basilar artery in our study, the success recanalization rate was not significantly different between these two locations (92.6% vs. 90%, *p* = 0.57), and the occlusion length was not a risk factor for successful recanalization or peri-procedural complications. In one case with intracranial vertebral artery occlusion, the occlusion length was 23.5 mm even though the recanalization was not successful. However, the occlusive length is an important reference for the use of long opening tools (especially long stents). It is speculated that the vertebral artery occlusion that is difficult to open is not caused by a long segment of atherosclerosis. The atherosclerosis is short in most patients, but the long segment of occlusion is probably caused by secondary thrombi which can be easily opened and compressed by a stent. This is probably because fewer side and perforating branches in the intracranial vertebral artery result in blood flow retardation proximal to the occlusion and subsequent thrombus formation, which leads to longer segments of occlusion in the vertebral artery.

In our study, telescoped stents were used in 29 (46.0%) patients while a long Enterprise stent (28 mm or 37 mm) was used in 44 (69.8%) patients. The reason is that the vertebral artery occlusion is long with many local thrombi. We prefer to use a closed-cell long stent (such as the Enterprise stent), which is beneficial for subsequent operations. In the use of closed-cell self-expanding stents, we should try not to use overlapped stents in order to reduce the time and complexity of the operation and subsequent occurrence of complications. Thus, a long stent is often used. In the need of overlapped or telescoped stents, it is better to place a balloon-expandable stent at the proximal end of a deployed self-expandable stent, which is beneficial to maintaining the opening and adherence of the overlapped parts of the stent. This is also helpful for endovascular devices to pass through the overlapped stents in case of complications and symptomatic restenosis. In our study, 29 (46.0%) patients were treated with the Enterprise stent combined with a balloon-expandable stent.

In recanalizing chronic occlusion of intracranial arteries, the most common complications are dissection and perforation (23), leading to a poor prognosis. In nine patients with basilar artery occlusion treated by Dashti et al. (14), dissection occurred in two cases, stent thrombosis in one case, and perforation in one case during the perioperative period. In nine patients with chronic basilar artery occlusion treated by Aghaebrahim et al. (15), arterial dissection took place in one case and perforation in one case, resulting in severe disability in one patient and death in the other. The main reason for these severe consequences is that stent angioplasty may severely damage important local perforating and side branches of the basilar artery, leading to severe brain stem function injury. Appropriate selection of patients with occluded arteries involving no perforating branches may decrease the rate of severe complications. In our study, dissection occurred in seven (19.3%) patients (five cases had vertebral artery occlusion and two cases had basilar artery occlusion). In total, two (2.27%) patients with dissection in the basilar artery died probably because of injury to the important functional perforating branches of the basilar artery, whereas five (5.68%) patients with intracranial vertebral artery dissection had better prognosis after appropriate management probably because the vertebral artery does not have so many important perforating branches such as the basilar artery. In the study by Aghaebrahim et al. (15) with nine vertebral artery occlusions, two patients with vertebral artery dissection also had a good prognosis (0 mRS in one patient and 1 in the other patient).

Our study found that patients with intracranial vertebral artery occlusion had better complications and follow-up outcomes. In addition to the abovementioned complications, there was only one death in the vertebral artery but four deaths in the basilar artery occlusion in the peri-procedural period though the difference was not significant. Moreover, the 6-month mRS scores were also significantly better in patients with vertebral artery occlusion than those with basilar artery occlusion. Nonetheless, patients with vertebral artery occlusion had a significantly greater occlusion length than those with basilar artery occlusion (21.11 ± 7.9 mm vs. 8.88 ± 3 0.50 mm, *p* < 0.0001). Patients with intracranial vertebral artery occlusion tend to have relatively mild brainstem infarction, which is often caused by low perfusion, and recanalization at the vertebral artery can increase brainstem blood perfusion without causing dissection of the basilar artery and subsequent brainstem injury. Moreover, the basilar artery has rich side and perforating branches, which may be easily injured by endovascular recanalizing operation. This may account for the better outcomes of recanalizing the vertebral artery occlusion, and it may also indicate that endovascular recanalization is safer and more effective in the vertebral artery than in the basilar artery. These rich arterial branches in the basilar artery may necessitate the appropriate selection of the indications for endovascular recanalization and careful operation during the recanalization procedure.

In our study, in-stent restenosis occurred in 25.3% (19) of patients at follow-up, which is greater than that of 17.6% in a study evaluating the safety and efficacy of the Enterprise stent in the treatment of severe symptomatic basilar artery atherosclerosis stenosis in 35 patients with over 80% stenosis (24). In this study by Zhao et al. (24), the main perioperative complication and the mortality rate were 0 while the in-stent restenosis rate was 17.6% at follow-up of over 6 months. Nonetheless, another study with the Wingspan stent to recanalize intracranial atherosclerotic stenosis reported a similar in-stent restenosis rate of 24.6% (25). In this study enrolling 77 patients with 79 total target stenosis >60% (25), the 30-day transient ischemic attack/stroke and death rates were 5.3 and 0%, respectively, and the cumulative transient ischemic attack/stroke and death rates were 8.1 and 0%, respectively, at a mean follow-up of 18.9 months (range, 12–23 months). At 3 to 24 months (median, 12 months), the in-stent restenosis was 24.6%, and rapid balloon inflation and longer length of stenosis were independent risk factors for the restenosis. In our study, blunt occlusion was a significant independent risk factor for successful recanalization, peri-procedural complications, and poor mRS outcomes at follow-up, whereas post-dilatation was a significant independent risk factor for in-stent restenosis and 1-year stroke and death events.

Some limitations existed in this study, including the retrospective and one-center study design, only Chinese patients enrolled, a small cohort of patients, no comparison with symptomatic non-acute occlusion in the anterior circulation, and no randomization, which may all affect the generalization of the outcomes. In the future, large controlled, randomized, controlled studies with multiple medical centers, and races involved will be needed to confirm the findings of this study.

In conclusion, endovascular recanalization for symptomatic non-acute occlusion of the vertebrobasilar artery is feasible even for a long occlusion, with a high recanalization rate, a low complication rate, and a good prognosis. Blunt occlusion and duration from onset to recanalization may affect successful recanalization, peri-procedural complications, and poor outcomes, while post-dilatation may affect in-stent restenosis and prognosis. Considering it is a procedure with a high risk of adverse effects, patients should be counseled accordingly.

## Data availability statement

The original contributions presented in the study are included in the article/supplementary material, further inquiries can be directed to the corresponding authors.

## Ethics statement

The studies involving human participants were reviewed and approved by the Ethics Committee of Henan Provincial People’s Hospital. The patients/participants provided their written informed consent to participate in this study.

## Author contributions

TL and LZ: study design. ZZ, LW, MG, ZM, YL, JQ, and BG: data collection. ZZ and BG: data analysis. ZZ: supervision. ZZ, LW, MG, ZM, YL, JQ, and BG: validation. All authors contributed to the article and approved the submitted version.

## Conflict of interest

The authors declare that the research was conducted in the absence of any commercial or financial relationships that could be construed as a potential conflict of interest.

## Publisher’s note

All claims expressed in this article are solely those of the authors and do not necessarily represent those of their affiliated organizations, or those of the publisher, the editors and the reviewers. Any product that may be evaluated in this article, or claim that may be made by its manufacturer, is not guaranteed or endorsed by the publisher.

## References

[1] Duan H Chen L Shen S Zhang Y Li C Yi Z. Staged endovascular treatment for symptomatic occlusion originating from the intracranial vertebral arteries in the early non-acute stage. Front Neurol. (2021) 12:673367. doi: 10.3389/fneur.2021.673367, PMID: 34220682 PMC8245001

[2] He X Zhang L Yang J Zheng H Li K Liu Y. Multimodal therapy for non-Superacute vertebral basilar artery occlusion. Interv Neurol. (2017) 6:254–62. doi: 10.1159/000477626, PMID: 29118803 PMC5662983

[3] Llopis G Quinones S Konschake M Simon De Blas C Hernandez LM Abramovic A. Atheromatosis of the brain-supplying arteries: circle of Willis, basilar, vertebral and their branches. Ann Anat. (2022) 243:151941. doi: 10.1016/j.aanat.2022.151941, PMID: 35378255

[4] Luo W Huang W Zhang M Liu X Guo Z Zhou P. Endovascular intervention for basilar artery occlusion in the elderly. Ther Adv Neurol Disord. (2021) 14:175628642110004. doi: 10.1177/17562864211000453 PMC8047973 33912242

[5] Machado M Borges de Almeida G Sequeira M Pedro F Fior A Carvalho R. Percutaneous transluminal angioplasty and stenting in acute stroke caused by basilar artery steno-occlusive disease: the experience of a single stroke Centre. Interv Neuroradiol. (2022) 28:547–55. doi: 10.1177/15910199211051830, PMID: 34704502 PMC9511620

[6] Tao C Li R Zhu Y Qun S Xu P Wang L. Endovascular treatment for acute basilar artery occlusion: a multicenter randomized controlled trial (ATTENTION). Int J Stroke. (2022) 17:815–9. doi: 10.1177/17474930221077164, PMID: 35102797

[7] Zhao W Zhang J Meng Y Zhang Y Zhang J Song Y. Symptomatic atherosclerotic non-acute intracranial vertebral artery Total occlusion: clinical features, imaging characteristics, endovascular recanalization, and follow-up outcomes. Front Neurol. (2020) 11:598795. doi: 10.3389/fneur.2020.598795, PMID: 33312156 PMC7703109

[8] Caplan LR. The intracranial vertebral artery: a neglected species. The Johann Jacob Wepfer award 2012. Cerebrovasc Dis. (2012) 34:20–30. doi: 10.1159/000339629, PMID: 22759402

[9] Gorelick PB Wong KS Bae HJ Pandey DK. Large artery intracranial occlusive disease: a large worldwide burden but a relatively neglected frontier. Stroke. (2008) 39:2396–9. doi: 10.1161/STROKEAHA.107.505776 18535283

[10] Wang Y Zhao X Liu L Soo YO Pu Y Pan Y. Prevalence and outcomes of symptomatic intracranial large artery stenoses and occlusions in China: the Chinese intracranial atherosclerosis (CICAS) study. Stroke. (2014) 45:663–9. doi: 10.1161/STROKEAHA.113.003508, PMID: 24481975

[11] Lindsberg PJ Soinne L Roine RO Tatlisumak T. Options for recanalization therapy in basilar artery occlusion. Stroke. (2005) 36:203–4. doi: 10.1161/01.STR.0000153796.49137.e8 15637313

[12] Hawkes MA Blaginykh E Ruff MW Burrus T Wijdicks EFM Rabinstein AA. Long-term mortality, disability and stroke recurrence in patients with basilar artery occlusion. Eur J Neurol. (2020) 27:579–85. doi: 10.1111/ene.14126, PMID: 31721389

[13] Ma L Liu YH Feng H Xu JC Yan S Han HJ. Endovascular recanalization for symptomatic subacute and chronic intracranial large artery occlusion of the anterior circulation: initial experience and technical considerations. Neuroradiology. (2019) 61:833–42. doi: 10.1007/s00234-019-02205-0, PMID: 31044262

[14] Dashti SR Park MS Stiefel MF McDougall CG Albuquerque FC. Endovascular recanalization of the subacute to chronically occluded basilar artery: initial experience and technical considerations. Neurosurgery. (2010) 66:825–32. doi: 10.1227/01.NEU.0000367611.78898.A3, PMID: 20190661

[15] Aghaebrahim A Jovin T Jadhav AP Noorian A Gupta R Nogueira RG. Endovascular recanalization of complete subacute to chronic atherosclerotic occlusions of intracranial arteries. J Neurointerv Surg. (2014) 6:645–8. doi: 10.1136/neurintsurg-2013-010842, PMID: 24249733

[16] Bozkurt B Aguilar D Deswal A Dunbar SB Francis GS Horwich T. Contributory risk and management of comorbidities of hypertension, obesity, diabetes mellitus, hyperlipidemia, and metabolic syndrome in chronic heart failure: a scientific statement from the American Heart Association. Circulation. (2016) 134:e535–78. doi: 10.1161/CIR.0000000000000450 27799274

[17] Muntner P Whelton PK Woodward M Carey RM. A comparison of the 2017 American College of Cardiology/American Heart Association blood pressure guideline and the 2017 American Diabetes Association diabetes and hypertension position statement for U.S. adults with diabetes. Diabetes Care. (2018) 41:2322–9. doi: 10.2337/dc18-1307, PMID: 30150235 PMC6196827

[18] Vederhus JK Rysstad O Gallefoss F Clausen T Kristensen O. Assessing alcohol use and smoking among patients admitted to the medical ward. Tidsskr Nor Laegeforen. (2015) 135:1251–5. doi: 10.4045/tidsskr.14.0848, PMID: 26269066

[19] Newcommon NJ Green TL Haley E Cooke T Hill MD. Improving the assessment of outcomes in stroke: use of a structured interview to assign grades on the modified Rankin scale. Stroke. (2003) 34:377–8. doi: 10.1161/01.STR.0000055766.99908.58, PMID: 12574545

[20] Zaidat OO Klucznik R Alexander MJ Chaloupka J Lutsep H Barnwell S. The NIH registry on use of the wingspan stent for symptomatic 70-99% intracranial arterial stenosis. Neurology. (2008) 70:1518–24. doi: 10.1212/01.wnl.0000306308.08229.a3, PMID: 18235078 PMC3506389

[21] Mori T Mori K Fukuoka M Honda S. Percutaneous transluminal angioplasty for total occlusion of middle cerebral arteries. Neuroradiology. (1997) 39:71–4. doi: 10.1007/s002340050370 9121654

[22] Gao P Wang Y Ma Y Yang Q Song H Chen Y. Endovascular recanalization for chronic symptomatic intracranial vertebral artery total occlusion: experience of a single center and review of literature. J Neuroradiol. (2018) 45:295–304. doi: 10.1016/j.neurad.2017.12.023, PMID: 29408529

[23] Kao HL Lin MS Wang CS Lin YH Lin LC Chao CL. Feasibility of endovascular recanalization for symptomatic cervical internal carotid artery occlusion. J Am Coll Cardiol. (2007) 49:765–71. doi: 10.1016/j.jacc.2006.11.029, PMID: 17306705

[24] Zhao Y Jin M Liu Q Liu D Chen J Du B. A long-term follow-up results of Enterprise stent in treatment of severe symptomatic basilar artery atherosclerotic stenosis. Zhonghua Nei Ke Za Zhi. (2016) 55:372–6. doi: 10.3760/cma.j.issn.0578-1426.2016.05.008, PMID: 27143187

[25] Shin YS Kim BM Suh SH Jeon P Kim DJ Kim DI. Wingspan stenting for intracranial atherosclerotic stenosis: clinical outcomes and risk factors for in-stent restenosis. Neurosurgery. (2013) 72:596–604. doi: 10.1227/NEU.0b013e3182846e09 23277374

